# Supplementary material for: A simple Turing reaction–diffusion model explains how PLK4 breaks symmetry during centriole duplication and assembly
Source: PLoS Biol. 2023 Nov 20;21(11):e3002391. doi: 10.1371/journal.pbio.3002391 (PMC10659181; doi:10.1371/journal.pbio.3002391)
Supplement: Attachment — Submitted filename: 133955_1_rebuttal_2706212_rw8n76.pdf [file pbio.3002391.s009.pdf]

Full Revision

Review COMMONS

Manuscript number: RC-2023-01864
Corresponding author(s): Jordan, Raff

1. General Statements [optional]

We thank the Reviewers for their detailed and constructive comments. As we describe below, we have now amended the manuscript to address their concerns and suggestions.

2. Point-by-point description of the revisions

Reviewer #1

In the first paragraph the reviewer states that our study is well presented and convincing, but that it seems "an incremental advance to the previous ones, which properly accounted for PLK4 symmetry breaking and are based on similar assumptions". We apologise for not explaining properly why our work is an important advance on these previous studies. Although both previous models can account for some aspects of PLK4 symmetry breaking, they both have significant issues. For example, Takao et al. perform no analysis of the robustness of their model, and from the small number of simulations shown it is clear that some *very* odd behaviours emerge—e.g. the oscillation of the dominant PLK4 site around the 6 compartments (Figure 3C, Example 3) and the bizarre manner in which PLK4 overexpression drives the formation of multiple PLK4 peaks (Figure 4B, first two examples). The authors do not comment on, analyse, or explain these strange phenomena. This model also relies on STIL being added to the system only after PLK4 has already broken symmetry; this is not plausible in rapidly dividing systems such as the fly embryo where Ana2/STIL levels remain constant through multiple rounds of centriole duplication (Steinacker et al., *JCB*, 2022). The Leda et al. model predicts that inhibiting PLK4 kinase activity will *deplete* PLK4 from the centriole, but it is now clear that PLK4 *accumulates* at centrioles when its kinase activity is inhibited (e.g. Yamamoto and Kitagawa, *Nat. Comms.*, 2019). Moreover, this model supposes no spatial relationship between PLK4-binding compartments; this has important implications for the system's behaviour (see point 1 in our response to Reviewer #2), and is biologically highly implausible. Thus, neither of the previous models can properly account for several important aspects of PLK4 symmetry breaking.

Moreover, the two previous studies are not based on similar assumptions. It is only through our analysis that we discover that the underlying biological process driving symmetry breaking in both previous models can be described in the same terms: with short-range activation and long-range inhibition causing diffusion-driven instability. This crucial conclusion was not obvious from, nor claimed by, either of the previous publications. We believe this is an important step in model development for these systems.

The reviewer raises a number of minor concerns, the first of which is a previous study from Chau et al. (Cell, 2012), which studies how two component systems break symmetry. Differential diffusion is not essential for symmetry breaking in some of the models considered by Chau et al., and so they wonder if it is really essential in our system. We thank the reviewer for pointing us to this study. It can be proven mathematically that differential diffusion is essential for symmetry breaking in the Turing-type framework. In the systems studied by Chau et al., symmetry can be broken without differential diffusion if one of the two components can be depleted from the cytoplasm. Such cytoplasmic depletion does not occur in traditional Turing-type systems, and it is almost certainly not occurring during PLK4 symmetry breaking—e.g. FRAP experiments show that PLK4 continuously turns over at centrioles (Cizmecioglu et al., *JCB*, 2010; Yamamoto and Kitagawa, *Nat. Comms.*, 2019). We discuss this point (p8, para.3).

The reviewer states that it is unclear which term in equations (3-4) and (5-6) correspond to the self-activation and activation/inhibition of the other component that are indicated in the schematic summary of the models shown in Figure 1C. As we now clarify, in general it is not always possible to pinpoint a single term in an equation that corresponds to activation/inhibition. Mathematically, a positive feedback for A means that $\frac{\partial f}{\partial A} > 0$, and a negative feedback for I means that $\frac{\partial g}{\partial I} < 0$. Hence, activation and inhibition can change depending on the values of these derivatives during the dynamics as these inequalities may be achieved with complex expressions that extend beyond the usual proportional relationships. We have amended the manuscript to make this clearer (p10, para.2).

The reviewer pointed out an error in the arrows in Figure 2 (we believe this is actually Figure 4). We thank the reviewer for pointing this out and have now corrected this mistake.

Reviewer #2

Major Comments:

1. The reviewer points out that in all models of PLK4 symmetry breaking the overexpression of PLK4 should be able to generate multiple PLK4 peaks (as, experimentally, PLK4 overexpression can generate up to 6 procentrioles around the mother centriole). The Reviewer suggests that the two previous models can do this, but we only show examples where PLK4 overexpression generates two peaks, and the reviewer questions whether this is a general limitation that would invalidate our approach. We are grateful to the reviewer for pointing this out, and we now expand our analysis and discussion of this important issue (p13-15). It is indeed possible to produce more peaks in our model using different parameters—e.g. decreasing diffusivity leads to thinner peaks, allowing more peaks to form (Figure 3B, Figure 5B). Importantly, however, when diffusion is decreased, the region of the parameter space in which only a single peak will form inevitably becomes

smaller—as diffusion can no longer efficiently suppress the formation of additional peaks around the rest of the centriole surface. Hence, in both our original models we struggled to find a parameter regime in which PLK4 robustly formed a single peak, but also formed >3 peaks when PLK4 was overexpressed. As we now discuss in detail, we believe that this is a general problem, as any model of PLK4 symmetry breaking must involve information being communicated around the centriole surface. We now show that a possible solution to this problem is to postulate that increasing PLK4 levels leads to a decrease in PLK4 diffusivity (Figure 3C, Figure 5C)—a biologically plausible possibility (p15, para.2).

In addition, it is not correct to say that the previous formulations of these models do not have this problem (or, in the case of Leda et al., the model actually has a related problem). This problem *must* apply to the Takao et al. model, as it also relies on information travelling around the centriole surface. This problem is far from obvious, however, because Takao et al. do not analyse the robustness of their model. This problem does not apply to the Leda et al. model, but this is because their model supposes no spatial relationship between the individual compartments and instead assumes that communication between compartments is instantaneous. This allows their system to overcome this communication problem and so robustly form a single peak at low PLK4 concentrations, while forming multiple peaks at high concentrations (as shown in Figure 6B). However, this requires that diffusion is sufficiently fast that concentration gradients are negligible between centriolar compartments, but not so fast that the relevant species are diluted in the much larger cytoplasm. It seems implausible that both of these effects may be achieved with a single diffusion rate in the real-world physical system.

2. The reviewer points out that in our modelling any multiple PLK4 peaks formed will tend to be evenly spaced around the centriole surface whereas, in their original formulations, the two previous models predict that any multiple 'winning' PLK4 compartments will not have any preferential spatial location with respect to each other. They ask that we address this difference and justify why we think our prediction is a better representation of PLK4 symmetry breaking. Although it is not obvious, neither of the previous models makes clear predictions about the spacing of multiple PLK4 peaks. As described above, Leda et al. assume no spatial relationship between PLK4-binding compartments, so relative peak-spacing cannot be assessed. Moreover, from the limited analysis shown, it is not clear that Takao et al. predict random spacing. The authors show only two simulations of PLK4 overexpression (Figure 4B, first two simulations) and the behaviour of PLK4 is very odd: the initial noise in the system fades away before PLK4 levels rapidly and near-simultaneously rise at multiple, reasonably well-spaced, peaks, before fading away to low levels—even after STIL addition. At the end of the simulation the "winning" compartments contain very low levels of PLK4 (often lower than the noise initially introduced into the system), but these compartments are reasonably (simulation 1) or very (simulation 2) evenly spaced.

Nevertheless, the reviewer is correct that the even spacing of multiple peaks is a feature of our model. Unfortunately, it is not possible to compare this prediction to reality because the spacing of multiple PLK4 peaks in cells overexpressing PLK4 has not been quantified yet. Thus, one has

to interpret published images, some of which support equal spacing while others do not (e.g. Kleylein-Sohn et al, *Dev. Cell*, 2007). Moreover, this analysis is likely to be complicated because CEP152 can form incomplete rings. This can be appreciated in Figure 2C in Hatch et al., (*JCB*, 2010) where the extra centrioles induced by PLK4 overexpression do not appear to be evenly spaced around the centriole, but are quite evenly spaced around the partial CEP152 ring. Therefore, equal spacing of peaks in ideal conditions is a feature predicted by our model that still needs to be fully explored experimentally. We believe that part of the power and value of our model is to suggest such hypotheses. We now discuss this important point (p26, para.2).

3. The reviewer questions our attempt to discretise our continuum model (where we convert the continuous centriole surface to a series of discrete compartments on the centriole surface and show that symmetry breaking can still occur). They note that we only show one example (9 compartments), they ask for more information about how the discretisation was done, and they question the independence of the compartments as PLK4 appears to accumulate in compartments adjacent to the dominant compartment. We apologise for the lack of clarity here. We now state that our models can break symmetry provided that there are at least two compartments, and we now include simulations showing that this happens for 2 – 10 compartments (Figure S2). The discrete model is a finite-difference discretisation of the continuum model (described in Appendix V). We also now clarify that the compartments are ‘independent’ in the sense that all chemical reactions only occur between components that are within the same compartment. The compartments are still spatially linked via a discretized diffusion (as would likely be the case at the centriole), which explains the observed relationship between neighbouring compartments.

4. The reviewer asks whether all the parameter values that satisfy the mathematical constraints we calculate for our models will break symmetry. If so, they suggest we are using a circular argument when demonstrating that the models break symmetry as we use parameter values chosen specifically to satisfy these constraints. In Turing-systems, one can mathematically calculate parameter constraints that allow symmetry breaking. As we now clarify, all parameters that satisfy these constraints *can* break symmetry, while any parameters outside these constraints *cannot* break symmetry. Thus, it was never our intention to claim something new or surprising when we illustrated the symmetry-breaking properties of our models (Figures 2 and 4, and associated parameter space analysis in Figures 3 and 5), so we apologise that our intention on this point was unclear. Rather, these Figures illustrate the detailed behaviour of each system under different conditions—something that is not possible to intuit from the equations alone.

5. The Reviewer requests more information about how we chose the particular parameter values we use to illustrate each model and asks that we convince readers that other sets of values that satisfy the derived mathematical requirements would result in the same qualitative outcomes. As described in point 4 above, and as we now state more clearly, it is a mathematical fact that parameter values that satisfy the derived mathematical requirements can

break symmetry. We now discuss our reasons for choosing specific parameters in more detail (see point 6, below).

6. The Reviewer asks whether the dimensionless parameters we use in our models have any biological relevance, and requests a biological interpretation of all of them. They also request that we relate the Diffusivity ratios of the Activator and Inhibitor species (D_I/D_A) to the experimental observations made by Yamamoto and Kitagawa. Relating our dimensionless parameters to biologically-relevant dimensional parameters is a complex issue. For example, one can see from equations (5) and (6) that simultaneously doubling (*A*), (*I*), and (*a*), and decreasing (*b)* by a factor of 4 leaves the system unchanged. Since the concentrations of *A* and *I* are unknown at the centriole surface, this means that it is not possible to determine the dimensional values of the rate of production of *I* (*a*) and its rate of conversion to *A* (*b*). This limitation is the root of the mathematical fact that FRAP experiments can reveal "off" rates but not "on" rates. Moreover, to convert the rate of loss of *A* (*c*) and *I* (*d*) into dimensional parameters it is necessary to know the timescale of symmetry-breaking. This is unknown, but was assumed to be on the order of hours in the previous models. This corresponds to a degradation/loss rate of minutes with our current choice of parameters, which is consistent with FRAP data (e.g. Yamamoto and Kitagawa, *Nat. Comms.*, 2019). Regarding the D_I/D_A ratio, the effective diffusion in our model depends on both the bulk diffusion and the binding/unbinding/degradation rates – a complexity also noted by Yamamoto and Kitagawa. This makes it very difficult to relate the "effective" surface diffusivity to the bulk diffusivity. We are currently investigating the form of this dependency, but this is a complex mathematical problem that is beyond the scope of this manuscript. These issues are difficult to discuss succinctly, so we now simply state that we chose specific parameter values based, in part, on the values and ratios used in the previous modelling papers (p10, para.2; p17, para.2).

Unfortunately, we could not find any experimental measurements of diffusivity in the Yamamoto and Kitagawa paper, as the Reviewer suggests. We now clarify, however, that the D_I/D_A ratio we use in both models (2500) is chosen to be between the *effective* diffusivity ratio (as the previous models used binding/unbinding rates rather than diffusivity) used by Takao et al. (10000) and Leda at al. (200). We also include a phase diagram showing how varying the diffusivity of both factors influences symmetry breaking in both models (Figure 3B, Figure 5B), and we state that we have chosen all remaining parameter values to reflect the parameter values in the original models, when adjusted to the same timescale.

7. The Reviewer asks for more information about how we normalised time in our simulations and whether the time in different simulations is comparable. We now clarify that the simulations run for a single unit of dimensionless time (so they can be compared), and that the reaction/diffusion parameters in the system are sufficiently large by comparison with unity that all simulations achieve steady state within a unit of time (p11, para.2).

8. The Reviewer asks whether concentrations of A and I can be compared between simulations, and also questions our description of A being uniformly accumulated in

Figure 4D, rather than uniformly depleted. We clarify that concentrations can be compared within a model, but not between models. This is because the dimensional values depend on the dimensional reaction rates, which differ between the models. This is not just a theoretical limitation; experimental fluorescence signals are typically compared in relative arbitrary units so the absolute values of different systems cannot be easily compared for the same reason. We agree with the reviewer that it is better to describe Figure 4D as showing uniform depletion of the activator, and we have adjusted the legend accordingly.

The reviewer makes a number of minor points that are not numbered.

The reviewer asks for clarification of what we mean by "robustness": does this refer to the ability to produce the same result in multiple simulations, or to the ability to produce the same result when parameter values are varied? If the latter, then the reviewer suggests our models are not very robust. We apologise for this confusion and now more clearly define what we mean by robust (p13, para.2). As we discuss in point 1 of our response to this Reviewer, our initial models are indeed not very robust at producing a single PLK4 peak over a range of PLK4 concentrations. We now discuss why this lack of robustness is likely to be intrinsic to any PLK4 symmetry breaking system, and how robustness in all such models can be improved by allowing diffusivity to vary with PLK4 expression levels (p13-p15).

The Reviewer points out that the original models introduce a noise term at every iteration, whereas we only introduce an initial noise term; they ask us to discuss this difference. We have run simulations introducing a noise term at every iteration and find that this makes negligible difference (Reviewer Figure 1, attached to the end of this letter). We do not take this approach, however, as this would significantly complicate the mathematical analysis that we perform (the additional noise term turns the system of PDEs into a system of SDEs which do not fit the Turing framework as readily). We now mention this in Appendix V.

The Reviewer states that the reaction schemes are unnecessarily repeated in Figures 1, 2 and 4. We would like to keep these schematics, as in Figure 1 we show a generic scheme (illustrating the two possible Turing-type reaction diffusion systems) whereas in Figures 2 and 4 we show specific reaction regimes (specifying the relevant species) that we test in each model. We feel this information will be useful to readers in this visual format.

The Reviewer states that it is confusing that we refer to the specific reaction parameters (k11 and k12) that need to be swapped to convert the Leda et al. model to the Takao et al. model, as this information will not mean anything to readers who are not familiar with the models. We agree and have now removed this information.

The Reviewer suggests several textual amendments and/or corrections. We thank the reviewer for spotting these and have amended them all accordingly.

Finally, the Reviewer states in their significance summary that although our key conclusions are convincing, they are not new as Takao et al. describe their model as analogous to a "reaction-diffusion system (also known as a Turing model)". We were aware that Takao et al. make this statement, but this does not invalidate the novelty or significance of our work. This is because although Takao et al. described their model as being *analogous* to a "Turing model", it is not actually a reaction-diffusion system, and it does not exhibit the property of long-range inhibition that is central to all Turing-systems to produce a single PLK4 peak. Instead, they use lateral inhibition (in which the influence of the inhibiting species does not extend beyond the neighbouring compartments) to reduce the number of potential PLK4 binding sites from ~12 to ~6. A single winning site is subsequently selected when STIL is added to the system—with additional positive feedback (not involving reaction-diffusion) ensuring that the compartment with most PLK4 becomes the dominant site. Their analysis of the reaction-diffusion version of their system is limited to a single supplementary figure (Figure S2D), and they do not perform or refer to *any* of the relevant mathematical analyses of their model that makes these well-studied systems such powerful tools. We believe that the model presented here is simple enough to draw the attention of the applied mathematics community while robust and complete enough to provide a mechanistic explanation of many interesting features and suggest new possible phenomena. We now discuss these points (p22, para.1).

Reviewer #3

The Reviewer found our manuscript well-written, and judged it of interest to centriole duplication enthusiasts. We interpret this to mean that the Reviewer did not think it of more general interest. This seems a harsh assessment, as the precise one-for-one duplication of centrioles is generally considered to be one of the great mysteries of cell biology. It is now widely appreciated that robustly breaking PLK4 symmetry to form a single PLK4 peak is crucial to this process. Thus, our discovery that this process can be described using a well-studied mathematical framework that has already been applied to a vast range of biological processes is potentially of significance even to non-centriole enthusiasts.

The Reviewer made a number of specific comments:

Figure 1. The Reviewer felt the graphic in Figure 1A could be improved by combining it with Figure 1B, and noted that the centrioles look strange. We thank the reviewer for these suggestions and we have now rearranged this Figure. We also now clarify that the schematic depicts *Drosophila* centrioles, which are simpler than human centrioles.

Figure 2. The Reviewer suggests that to make the system depicted in Figure 2A fit as a Type I Turing system we have to assume that (I) must dissociate from the centriole or be degraded at higher rates than (I) converts (A) to (I). They suggest this assumption is implicit in the model and they request further explanation. The reviewer is correct that, in Model 1, the degradation/dissociation of (I) is the root of its self-inhibition. However, we do not

need to make any assumption about the relationship between the rate at which I converts A to I (*b)*, and the dissociation/degradation rate of I (*d)* for this system to work (as the Reviewer implies). This is because, whatever these rates are, the system will approach a steady-state where the production and degradation terms balance, and it is the stability/instability of this state that determines whether the system can break symmetry. Since the degradation rate of I (the -dI term in equation 4) increases more rapidly than its production rate (the $bA\sqrt{I}$ term in equation 4) as I increases, this results in a *stable* (i.e. self-inhibiting) system regardless of the parameter values. We have rewritten the sections explaining these equations to try to make these points more clearly and to point readers to Appendix II where we explain the form of the equations.

The Reviewer asks if in Model 1 it is realistic to assume no turnover or loss of PLK4 (A), and will the system still work if this is altered? This is a good point. In Model 1, we set c=0 as this makes the analysis significantly simpler, enabling us to display the mathematical predictions alongside the numerical simulation. We have now added the (*c*,*d*) phase diagram to show the effect of varying these parameters on the symmetry breaking properties of the system (Figure 3D). We find that the value of *c* has a relatively weak effect on the symmetry breaking properties of the model since it does not affect the function of A as an activator.

The Reviewer asks if our 1D model would work in 2D, and notes the PLK4 peaks in our models are broad, likely limiting the number of peaks formed. They also note that in our Model 1 it is the unphosphorylated form of PLK4 that accumulates in the peak, which seems unlikely as it is widely believed that PLK4 must be active to phosphorylate STIL to promote its interactions with SAS6 and CPAP. From a mathematical perspective, modelling our system in 2D would produce very similar results. Symmetry breaking is driven by long-range inhibition/short-range activation, and these behaviours will work analogously in 2D. As discussed in our response to Reviewer #2 (point 1), the broad peaks do indeed limit the number of centrioles that can form, but by altering the parameters we can generate more peaks that are less broad (Figures 3 and 5). The Reviewer is correct that Model 1 (based on Takao et al.) predicts that non-phosphorylated PLK4 (A) accumulates in the peak. This is also true of the original Takao et al. model, although this was not highlighted or commented on by the authors. We now expand our discussion of this point (p25-p26).

The Reviewer asks if our models can form multiple peaks at higher PLK4 levels. This is again related to Reviewer #2, point 1, and we now show that this is indeed possible under the appropriate parameter regime (Figure 3C and Figure 5C).

The Reviewer asks for more description of how lateral diffusion works in our system. For example, do we consider that not every molecule of (I) will diffuse laterally (as some will be lost to the cytoplasm), or that the probability of a molecule leaving the surface will increase as distance/time increases. We apologise for our lack of clarity. We now state that the proportion of molecules not rebinding to the surface is accounted for in the reaction components of all our models (p7, para.1). In reality, and as we now state, the relationship between this loss

and the diffusion rates (and their relation to distance/time, for example) is complicated. We are investigating this relationship in more detail, but this is beyond the scope of the current paper.

The Reviewer asks if symmetry breaking might eventually occur if the system in which we reduce the kinase activity of PLK4 (Figure 2D) were given more time. They also ask whether reducing PLK4 levels by half would lead to a failure in site-selection. The kinase inhibited scenario we show here will not break symmetry over any period of time; this can be proven mathematically, and is verified in the numerical simulations (Figure 3A and 5A, bottom left regions of graphs), which we now state more clearly are always run for a long enough period to reach a steady-state (p11, para.2). The effect of reducing PLK4 levels in our models is analysed in the phase diagrams shown in Figure 3 and 5 (and analysed in more detail in Figure S1), where it can be seen that there are multiple PLK4 concentrations that can be halved without a failure in site selection (although, see also our response to Reviewer #2, point 1).

The Reviewer pointed out some errors in our presentation of Figure 3, (and suggested some improvements in presentation in a point further below) and also asked for more information about the parameters used to generate the data in Figures 2B-D and 4B-D. We thank the Reviewer for these suggestions and have made these changes and provided the additional information requested (e.g. marking the specific parameters used in our simulations on the phase diagrams shown in Figure 3 and Figure 5 with *coloured dots*).

The Reviewer points out that when PLK4 levels and activity are both high no centrioles are produced in Model 2, whereas 1 centriole is produced in Model 1—neither of which are consistent with experimental observation. We now show an expanded parameter space (new Figures 3A and 5A) where it can be seen that this is not a problem for Model 1. For Model 2, the region of high kinase levels and activity (*dark blue*, top right, Figure 5A) corresponds to the uniform accumulation of the activator species. Thus, while there are no *peaks*, this region might produce multiple centrioles, as it is equivalent to a compartmental model in which all of the compartments are occupied. We now discuss this point (p19, para.1).

The Reviewer questions how the biology fits a Type II Turing system, pointing out that current data suggests that active PLK4 turns over more rapidly at centrioles, whereas in the Type II model we describe (based on the Leda et al. model) it is the phosphorylation state of STIL that determines which species of PLK4:STIL turns over rapidly. They also question the logic of the Model 2 Type II circuit (Figure 3A), questioning how A could drive the dephosphorylation of STIL to promote the production of I. We agree that current data is more consistent with phosphorylated species of PLK4 turning-over more rapidly at centrioles, but this is not what Leda et al. proposed, and so this is not what we implemented in trying to reformulate their model (although this is effectively the change we make that turns the Leda et al. model into the Takao et al. model). As to the second point, the Reviewer has correctly spotted a problem with our model that arises because the direction of the arrows linking A and I were inadvertently flipped in Figure 4A. This mistake has been corrected, and we now explain more clearly how the biology of this system fits a Type II Turing system in the legend.

The Reviewer points out that although we can convert the Leda et al. Model (Model 2) to the Takao et al. Model (Model 1) simply by changing the identity of the A and I species, the underlying assumption of the Takao et al. model (that non-phosphorylated PLK4 promotes its own accumulation) was not an inherent assumption of the Leda et al. model. We apologise for this confusion. As we now clarify (p20, para.1) the Reviewer is correct that when we make mathematical changes to the Leda et al. model we must also assume changes in the underlying biology—so that non-phosphorylated species of PLK4 are now slow diffusing, rather than non-phosphorylated species of STIL, as originally proposed). As the Reviewer points out, current data suggests that non-phosphorylated species of PLK4 do turnover more slowly, although it is not clear why—for example, liquid-liquid phase separation driving the formation of PLK4 condensates has been postulated, but is far from proven. This remains an interesting problem that will be further probed mathematically and experimentally.

Finally, we thank the Reviewers again for their helpful comments. We hope they agree that the changes we have made have addressed their main concerns and improved the manuscript so that it is now suitable for publication.

Yours sincerely,

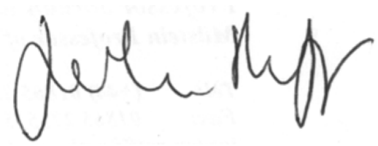

Reviewer Figure 1

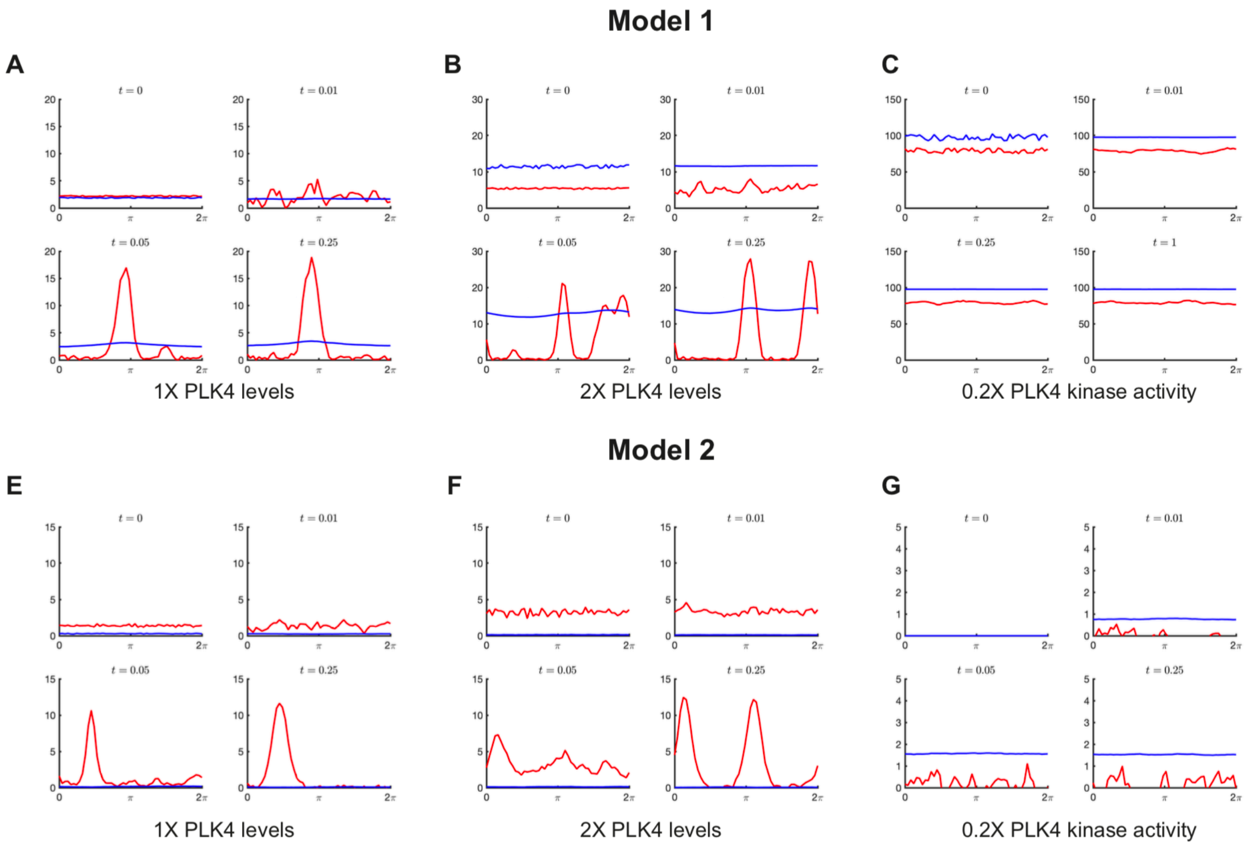

Graphs show the output from simulations of Model 1 (A-C) or Model 2 (E-G) under the same conditions as shown in Figure 2 (Model 1) and Figure 4 (Model 2), but where we introduce a noise term at every iteration, rather than at only the start of the simulation (as in the original Figures). This makes negligible difference to the final solutions under each condition, although obviously there is more noise in the final solution.
