# Supplementary material for: A simple Turing reaction–diffusion model explains how PLK4 breaks symmetry during centriole duplication and assembly
Source: PLoS Biol. 2023 Nov 20;21(11):e3002391. doi: 10.1371/journal.pbio.3002391 (PMC10659181; doi:10.1371/journal.pbio.3002391)
Supplement: Attachment — Submitted filename: Response to Reviewers.docx [file pbio.3002391.s011.docx]

**Response to Reviewers**

**Reviewer #1**

*The authors convinced me with their description of the novelty of the model and its significance with respect to previous models. I also read the authors' response to other reviewers and was also convinced that the description of a symmetry break in the Turing reaction diffusion mechanism may be of interest beyond the centriole duplication community.

I have considered the authors' argument about the novelty of their findings compared to the work of Chau et al. (Cell 2012). However, I still believe that the final reaction scheme they propose in Figure 4 was already predicted in that publication to break symmetry and polarise cells with two interacting components. The key to this mechanism was not cytoplasmic depletion but local concentration. I appreciate that in Chau et al the components do not permanently detach and relocalise, which PLK4 does, and so the components concentrate to complete cytoplasmic depletion, which PLK4 does not, but the interaction scheme is still the same. The dynamics are different, but the mechanism is the same. So for me, the conceptual innovation of this model is limited and the work of Chau and Lim could be discussed more than just in the introduction. However, it is valuable to apply it to the example of PLK4 dynamics.* We are slightly puzzled by the Reviewers comment on the novelty of our work in light of the Chau et al. paper. The schematic in Figure 4 is a typical representation of a classic Turing-system that has been known to break symmetry under the right conditions since the 1950’s. As we make clear throughout the manuscript (and in our title), our work is based entirely on this original framework. We never claim that this is a novel or conceptually innovative scheme, and so the fact that Chau et al. came up with a similar scheme in 2012 does not undermine our findings. We also think the Reviewer is incorrect when they state that the mechanism of our model and the Chau et al. model are the same. First, the arrows in our Figure 4 have a subtly different meaning to the arrows in the equivalent figures in Chau et al.; they represent the signs of the specific binding terms in Chau et al., while in our model the arrows represent the signs of the partial derivatives of the reaction terms evaluated at the steady state. Second, the Reviewer’s claim that our symmetry-breaking mechanism is the same as in the Chau et al. paper is not correct. Differential diffusion is not essential in the Chau et al. system, but it is at the core of the reaction-diffusion model that we study here.

In summary, the Chau et al. paper is an interesting and well-executed study that bears many similarities to the Turing-system we study and that we cite as such in the Introduction. Since the dynamics, physical set-up, and basic instability mechanisms are clearly different, however, it seems inappropriate to further cite this paper as providing the conceptual framework underpinning our work.

**Reviewer #2**

*In their opening paragraph these reviewers state that our work is of insufficient novelty or significance, primarily because our continuum reformulation of the previous models does not increase our understanding of the system, nor more accurately describe PLK4 symmetry breaking.* This is a very harsh assessment. We demonstrate that the reason existing models work is the basic underlying mechanism of “short-range activation/long range inhibition”, a crucial, and intuitively simple, explanation of how the PLK4 system actually works that is absent from both previous studies. Hence, our model is much more general and provides an overarching theoretical explanation on the basic required features of such models. Moreover, our models *do* provide a more accurate description of PLK4 symmetry breaking—correctly predicting what happens when PLK4 kinase activity is inhibited (in contrast to the Leda et al. model) without exhibiting any of the odd behaviours shown (but not explored or explained) in the simulations presented in the Takao et al., paper. Furthermore, by reformulating the system as a continuum model, we demonstrate that this principle of short-range activation/long range inhibition can be extended beyond the discrete models studied previously. This is likely to be important as two recent papers show that CEP152, the main PLK4 receptor, is not detectably organised into discrete binding sites at the centriole surface (Scott et al., *JCB, in press*; Sullenberger et al., *JCB, in press*), which is compatible with our model, but incompatible with the previous models. In summary, our model is more general (based on clear and simple general principle), simpler in formulation, mechanistic (identifying the physical mechanisms responsible for the observation) and has more predictive power. Therefore, we strongly believe that it is novel and significant as it will not only improve our understanding of these systems but also allows for direct quantitative fitting of new experiments.

The Reviewers then describe the major comments that they felt had not been addressed in the revision, first making two comments about our response to Reviewer #1.

*First, they requested that we explicitly spell out the problems with the Takao et al. model in the manuscript, rather than in our rebuttal letter, stating that this is crucial for readers to understand why we wanted to reformulate this model.* In the cell biology field, it would be very unusual to directly criticise another paper in this way, and we are reluctant to do so. The Takao et al. paper contained some important cell biology, and an interesting first attempt at modelling this system mathematically. It is certainly not deserving of overt criticism, although we believe the mathematics were quite preliminary and the simulation results were not explained or analysed in detail, which was perfectly reasonable for this type of paper (a mix of biology and maths). Moreover, any potential problems with the Takao et al. model were not the primary motivation for our work; we simply realised that PLK4 symmetry breaking could be explained using a Turing framework, and then used the underlying biology of the Takao et al., and Leda et al., models as a basis for formulating the Type I and Type II systems so that these previous models could be compared. We do make it very clear in the manuscript that neither of the previous models can fully explain PLK4 symmetry breaking, and would like to leave it at that.

*Second, the Reviewers thought our statement that the Leda et al. model predicts that inhibiting PLK4 activity leads to PLK4 depletion is wrong—thinking that this may be the case in our reformulation, but is not the case in the original model****.*** We were initially confused by this as we have talked extensively to Andrew Goryachev and Andrew Holland (the two senior authors on the Leda et al. paper), and there is no doubt that their original formulation gets this wrong. On careful re-reading of Leda et al. we think the confusion might arise because they state that “inhibition of PLK4 kinase activity produces in our model progressive *accumulation* of inactive PLK4 in the form of a symmetric ring surrounding the mother centriole” (our italics). This *accumulation* is technically true, as in the original formulation inhibited PLK4 is *added* to the system—so it will start to accumulate at the ring even though it does so to very low levels. In contrast, in our reformulation of the Leda et al. model, PLK4 is already bound to the centriole surface, so PLK4 inhibition leads to its *depletion*. The key point here is that the Leda et al. model (in either formulation) predicts that inhibited PLK4 accumulates to much *lower* levels than the uninhibited protein, whereas in reality it accumulates to much *higher* levels. This is a major problem for the Leda et al. model. We have now reworded this section to avoid any potential confusion.

The Reviewers then comment on the numbered points they raised in their original review.

*1. The Reviewers noted that to generate multiple peaks in the PLK4 overexpression limit we have postulated a mechanism in which Plk4 diffusivity around the torus decreases with cytoplasmic Plk4 concentration. As a result, the width of a Plk4 peak becomes smaller as the number of peaks increases. They state that this expanded analysis prompts several questions, which we repeat and answer below:*

*(a) How much thinner do Plk4 peaks become? In Model 1, the maximum number of peaks that can be achieved robustly seems to be 5 (and in some cases 6, Figure 3B).* As we now clarify, peak width depends on the specific parameters chosen, so it is possible to find parameters to make the peaks arbitrarily thin. In Figure 3B, the maximum number of peaks depicted is 6, but this limit is partially due to the finite resolution of the figure (zooming into the lower left corner will reveal infinitely many peaks), and partially due to the fact the figure is under conditions where all other parameter values are held constant. If we vary these other parameters appropriately one can make many more, thinner, peaks. It is therefore not a restriction of the model.

*(b) Is there a theoretical limit to the number of peaks formed?* The number of peaks is determined by how many peaks can “fit” on the ring for a given parameter regime. Since scaling all of the parameters could allow an indefinite number of peaks to fit, there is no *theoretical* limit, within the model, to how many peaks can form. Of course, at a certain point, the assumptions of the model break down and the model is not a valid representation of the system. As we now explain, the *practical* limit depends on the size of the ring and the lower bound of the diffusivity (p15, para.2).

*(c) If so, how does the maximum number of peaks relate to the 9 peaks in which Plk4 is reportedly coalescing upon inhibition of Plk4 kinase activity (mentioned in the Discussion on p. 27)? If there is not theoretical limit to the maximum number of peaks, why should there be only 9 peaks experimentally?* As described in our answer to (b), there is a practical limit on the number of peaks that can form under any parameter regime. Moreover, the new papers *in press* at JCB only report up to 9 PLK4 peaks when PLK4 kinase activity has been inactivated for many hours—so under extremely non-physiological conditions (as PLK4 kinase activity is essential for symmetry breaking). Nevertheless, this maximum of 9 peaks presumably reflects the underlying 9-fold symmetry of the centriole, which we discuss further in point (d) below.

*(d) More generally, could the 9 peaks observed upon Plk4 kinase inhibition invalidate the use of formulating Plk4 symmetry breaking as a Turing-based two-component reaction-diffusion system on a continuous line?* We agree that the 9-peak limit observed with inactive PLK4 presumably reflects the 9-fold symmetry of the centriole, suggesting that the centriole surface is not a perfect homogeneous continuum. At the very least, the 9-fold symmetry of the centriole appears to influence the number and positioning of the kinase-inhibited PLK4 peaks, although, as we argue in the point above, this process is very unlikely to be relevant to normal physiological symmetry breaking. Crucially, however, this observation does not invalidate our modelling for two reasons. First, a continuum does not necessarily have to be homogeneous, and the real system probably exists somewhere between the limits of discrete compartments and a homogeneous continuum (and the distribution of CEP152 reported in the JCB papers *in press* strongly supports this idea). Since all previous modelling was performed in the compartmental limit (which now seems unlikely to be correct), it is important to show that symmetry breaking is still possible if the surface is continuous, or lies somewhere between the continuous and discrete. Second, the most important conclusion from our work is not that Plk4 symmetry breaking takes place on a continuous surface. Rather, it is to show that the Turing framework is a simple and effective way to describe the system—whatever the underlying reactions and geometry—and that the same underlying biological principle of short-range activation/long-range inhibition can explain both previous models.

(*e) Can multiple peaks be generated in discretized versions of the model? And does the discretization allow for multiple peaks upon Plk4 overexpression without the requirement for the peaks to become thinner, or does the set compartment size prevent forming multiple peaks? This is an important point to clarify.* This is an important point and we apologise for not discussing this properly. In the discrete version of the model the “width” of the peaks is an undefined property since PLK4 either accumulates in a compartment or it doesn’t—so no variation in peak “width” is possible. We now emphasise more clearly, however, that our models behave essentially in the same way in either the continuum or discrete limits, and we now show that our models produce multiple peaks upon PLK4 overexpression and fail to break symmetry when PLK4 kinase is inhibited in the discrete limit (new Figure 6).

*(f) Furthermore, the authors state that the inability to form multiple peaks is a fundamental issue that would be present in any spatial model (bottom of p. 14), but state also that "it is not clear if these problems also occur in the original formulations of the Takao et al. model as no analysis of the robustness of the model to parameter changes was performed" (top of p. 14). Both Leda et al. and Takao et al. models form multiple peaks in overexpression conditions without peaks becoming thinner as their number increases. What convinces the authors that their mechanism is a better representation of the formation of multiple peaks upon Plk4 overexpression?* We think the Reviewers are making two points here. The first seems to be a suggestion that there is a discrepancy between our statements on p14. We apologise for any confusion, but the statement quoted at the top of page 14 was meant to be in reference to the ability of this model to robustly form a single peak—which, at the bottom of p14, we point out is not assessed in the original manuscript—rather than its ability to produce multiple peaks in the overexpressed limit. We have amended the text to clarify this. The second is essentially pointing out that, in the discrete limit, any multiple PLK4 peaks will be of the same width, whereas in the continuous limit multiple peaks will be thinner than a single peak; which model better describes the real situation? Unfortunately, the ‘real situation’ is not known, so we cannot assess whether the discrete or continuous limit better describes reality. Importantly, our models work at either limit (and anywhere in between, as we can easily discretise it on multiple compartments), whereas both previous models only work at the discrete limit.

*2. We thank the authors for adopting our suggestion to discuss the strong prediction their reformulation of the models makes on the spacing of multiple Plk4 peaks. However, we do not agree with their statement that this prediction cannot be compared to that of the Leda et al. model. In the rebuttal document, the authors correctly note that "Leda et al. assume no spatial relationship between PLK4-binding compartments." From this, the authors infer that the model does not make predictions about the spacing of multiple Plk4 peaks, stating that "peak-spacing cannot be assessed." Instead, we interpret that a strong prediction from the lack of spatial relationship in Leda et al. is that multiple peaks have no dependence on each other, and thus should be randomly positioned relative to each other.* We stand by the statement in our rebuttal. As the formulation of the Leda et al. model does not permit any conclusion other than that spacing is random, it seems disingenuous to deem this a *prediction* of the model, when in reality it is a core underlying *assumption* that the authors impose on the system. That result is therefore out of reach of the Leda et al. model. In contrast, the semi-regular peak spacing in our reaction-diffusion system is a higher-order effect that emerges from our model. It occurs due to small fluxes of material across the peaks that arise due to slight variations in the gradient. The Leda et al. model neglects such higher order effects *a priori* by supposing instantaneous transfer of material between compartments. We think it is unreasonable, and unhelpful to readers, to insist we compare the predictions made by our model to the underlying assumptions of the Leda et al. model as though they are the same thing.

***Points 4-6.*** *We asked the authors to clarify their parameter choices. From the rebuttal document and the revised manuscript, it remains unclear how the specific parameter values were chosen. In the revised manuscript, the authors mention that "values were chosen in part to reflect values and ratios in the previous modelling papers when adjusted to the same timescale", without specifying how. For the reader's interest, the authors should make it clear how values of the original models are mapped to specific parameter values in their reformulations, and specify which values in the reformulations originate from which values in the original models. This could be integrated for instance in the Appendix pertaining to the reformulated models. As stated already during our initial review, we think that the authors should discuss if any other set of values that satisfies the derived mathematical requirements would result in the same qualitative outcomes with similar robustness. Alternatively, potential empirical reasons why these values are preferred should be mentioned explicitly. Furthermore, it is unclear why the specific parameter values chosen are different for the two models. This is especially puzzling as it is then shown that the two models can be written in the same terms. Whether the qualitative outcome of the models is analogous when using the same parameters for both models are used should be mentioned. If this is not the case, an explanation should be provided.* We find these comments puzzling. The original Takao et al. models contained 10 parameter values, none of which were known and all of which were estimated. The original Leda et al., model contained 46 parameter values, none of which were known and all of which were estimated. Our models each contain 6 parameter values, none of which are known and all of which are estimated. As none of the parameters in any of the models are known, assigning an absolute value to any one of them is essentially meaningless—even if our guess is out by an order of magnitude, the model could produce the same result with the same robustness simply by appropriately scaling the other unknown parameters. Moreover, there is no reason that the parameter values should be the same between the two models. First, many of the parameters represent slightly different things between the models so a direct comparison would be inaccurate. Second, since the reality cannot be explained by both models (they are alternative, competing models), there is no theoretical or practical reason why both models must work within the same parameter regime. We have now tried to explain this more clearly in the text (p11, para.1) but, unless we are missing something, we feel this parameter issue is a complete red-herring.

*In response to our comments in the 'significance' section, in which we had noted that Takao et al. already mentioned that a Turing model can explain symmetry breaking at the centriole, the authors revised the manuscript by mentioning that "Takao et al. described their system as being analogous to a 'Turing model', but it is not a reaction-diffusion model" (p. 23). Here, the authors refer to the lateral-inhibition model, which is analogous to reaction-diffusion model, but indeed not one per se. However, as the authors themselves mention in the rebuttal document but not in the revised manuscript, Takao et al. also performed an analysis of the reaction-diffusion version of their model. Additionally, both the lateral-inhibition and the reaction-diffusion versions of the original Takao et al. model consisted of two components. Therefore, the fact that a two-component Turing reaction-diffusion model can explain how mother centrioles break PLK4 symmetry to generate a single daughter is already evident from Takao et al.'s work.* In the Takao et al. paper, the name “Turing” is mentioned precisely once: *“This mutual relationship between the active and inactive forms of Plk4 is similar to that of the reaction–diffusion (RD) system (also known as the Turing model) or its analogue, the LI system, which forms periodic patterns at the cellular or tissue level”*. As we noted in our original response, and as the Reviewers seem to concede in the text above, the Takao et al. model *is not a Turing-model*, the authors *never claim that it is a Turing model*, and they *do* *not once mention, discuss, or use any of the standard mathematical analysis procedures that are routinely used to analyse Turing systems*. As the Reviewers point out, we acknowledge that the analysis Takao et al. present in Figure S2 could be interpreted as being relevant to a Turing model, but it is not presented as such (Turing-models are not mentioned in the Figure or legend), and most biologists (and we suspect most mathematicians) would struggle to recognise which elements of this Figure examine Turing-like properties. We simply do not understand how the Reviewers conclude that the Takao et al. paper makes it self-evident that a two component Turing reaction-diffusion model can explain PLK4 symmetry breaking.

More generally, we acknowledge that the previous models are valuable and an important starting point, which is why we discuss them extensively and largely refrain from criticising them. Thanks to the ideas presented in these models, and more recent experiments, our work presents a unifying mechanistic view that is flexible to further generalisation and that captures well our current knowledge of the system.

**Reviewer #3**

*The Reviewer states that although our models still have some inconsistencies, we discuss these sufficiently, and so our revisions have largely addressed their main concerns. They still feel that our idea that PLK4 kinase activity may be required only for symmetry breaking and not for daughter centriole assembly is unlikely, and quote several observations that would seem to contradict such a model.* We agree that this is something of a heretical idea, and that the data the Reviewer quotes argue against it (although we feel they do not conclusively disprove it). We have now rewritten this section to adopt a more cautious tone.

We would like to thank the Reviewers again for taking the time to read and comment on our revised manuscript, and hope that you and the Reviewers will find the manuscript now suitable for publication in *PLoS Biology.*

We look forward to hearing from you.

Yours sincerely,

Professor Jordan Raff

César Milstein Chair of Cancer Biology
